# Evaluation of multi-segmental kinematic modelling in the paediatric foot using three concurrent foot models

**DOI:** 10.1186/1757-1146-6-43

**Published:** 2013-10-31

**Authors:** Ryan Mahaffey, Stewart C Morrison, Wendy I Drechsler, Mary C Cramp

**Affiliations:** 1 School of Health, Sport and Bioscience, University of East London, Stratford, London E15 4LZ, England; 2 Department of Allied Health Professions, Glenside Campus, University of West England, Blackberry Hill, Bristol BS16 1DD, England

**Keywords:** Foot, Paediatrics, Gait

## Abstract

**Background:**

Various foot models are used in the analysis of foot motion during gait and selection of the appropriate model can be difficult. The clinical utility of a model is dependent on the repeatability of the data as well as an understanding of the expected error in the process of data collection. Kinematic assessment of the paediatric foot is challenging and little is reported about multi-segment foot models in this population. The aim of this study was to examine three foot models and establish their concurrent test-retest repeatability in evaluation of paediatric foot motion during gait.

**Methods:**

*3*DFoot, Kinfoot and the Oxford Foot Model (OFM) were applied concurrently to the right foot and lower limb of 14 children on two testing sessions. Angular data for foot segments were extracted at gait cycle events and peaks and compared between sessions by intraclass correlation coefficient (ICC) with 95% confidence intervals (95%CI) and standard error of measurement (SEM).

**Results:**

All foot models demonstrated moderate repeatability: OFM (ICC 0.55, 95% CI 0.16 to 0.77), 3DFoot (ICC 0.47, 95% CI 0.15 to 0.64) and Kinfoot (ICC 0.43, 95% CI -0.03 to 0.59). On the basis of a cut-off of 5°, acceptable mean error over repeated sessions was observed for OFM (SEM 4.61° ± 2.86°) and 3DFoot (SEM 3.88° ± 2.18°) but not for Kinfoot (SEM 5.08° ± 1.53°). Reliability of segmental kinematics varied, with low repeatability (ICC < 0.4) found for 14.3% of OFM angles, 22.7% of 3DFoot angles and 37.6% of Kinfoot angles. SEM greater than 5° was found in 26.2% of OFM, 15.2% of 3DFoot, and 43.8% of Kinfoot segmental angles.

**Conclusion:**

Findings from this work have demonstrated that segmental foot kinematics are repeatable in the paediatric foot but the level of repeatability and error varies across the segments of the different models. Information on repeatability and test-retest errors of three-dimensional foot models can better inform clinical assessment and advance understanding of foot motion during gait.

## Background

Traditionally, the foot is presented as a single rigid segment in three dimensional (3D) kinematic modelling [1-3] but in recent years, multi-segment foot models have been developed. These models have advanced understanding of the multiple articulations and functional complexities of the foot during gait in typically developing children [4-6], adolescents [7], adults [8-12] and clinical populations [13,14]. Due to the choice of (available) multi-segment foot models, selection of a foot model for gait analysis is challenging with the utility of the model being determined by both functional features of model and repeatability of model outputs. In order to identify foot models appropriate for application with children, this research reports repeatability of three multi-segment foot models with varying functional features.

The functional features of multi-segment foot models relate to factors such as segmental definition, degrees of freedom and joint axis determination [15,16] and three models were selected that represented the range of reported foot models. The Oxford Foot Model (OFM) is a four segment model (shank, hindfoot, forefoot and hallux) that has been adapted by Stebbins *et al.*[4] for use in a paediatric population. Repeatability of OFM has been established in 15 typically developing children (mean age 9.5 years) with within-subject SD for maximal values of 3.0-8.4° reported at the hindfoot and forefoot segments. Curtis *et al*. [17] measured the repeatability of OFM at discrete data points over the gait cycle in typically developing children. The findings revealed intraclass correlation coefficients (ICCs) of -0.14-to-0.96 and typical error of measurement values of 0.93-8.56° for maximal, minimal and mean joint angles. However, OFM does not currently include a midfoot segment. Leardini *et al*. [8,10] developed 3D foot which is a five segment model (shank, calcaneus, midfoot, metatarsals and hallux) and allows for normalisation of joint angles to a standing position. Repeatability of 3DFoot has been reported for two adult subjects with average within-subject SD ranging from 0.9-1.3° for hindfoot, midfoot, and forefoot [18] but its use in the paediatric foot had yet to be reported. Kinfoot is a nine segment model (shank, hindfoot , two midfoot segments, two forefoot segments, two toe segments and a hallux) developed by MacWilliams *et al*. [7]. Between-subject repeatability for this complex model has been reported for 18 adolescents (mean age 12.4 ± 2.6 years) and ranged from 0.8-2.9° across all motions [7].

Indications are that multi-segment foot models can be applied successfully to paediatric feet, but with a limited number of studies available, there is a need to examine repeatability of established multi-segmental models in this group. Evaluation of the paediatric foot presents many challenges, particularly the smaller surface area for marker application and greater gait variability [19]. Improvements in motion capture technology have addressed some of the issues with marker tracking within a small area. To determine repeatability in a paediatric population, when inherent variability is relatively high, a protocol to reduce inter-trial variability is required. Previous research utilised concurrent marker sets from five whole-body models to analyse joint kinematics in adult gait [20]. The advantage of this approach is that inherent gait variability is accounted for when examining each model. Thus, the aim of this study was to examine three foot models and establish their concurrent test-retest repeatability in evaluation of paediatric foot motion during gait.

## Methods

Seventeen participants (age range 6–11 years) were recruited from a convenience sample of children from University staff and an after-school club. Participants were typically developing children and excluded if they presented with any medical conditions affecting neuromuscular or orthopaedic integrity of the foot or lower limb or any conditions leading to altered foot posture and/or gait changes. Consent was obtained from the parents of the children and assent from all participants. Ethical approval was granted by University Research Ethics Committee (Ref No. ETH/13/11).

### Protocol

A ten camera Vicon 612 (Vicon Motion Systems Ltd, Oxford, UK) system was used to capture reflective marker coordinates (sampling rate 100 Hz) within the capture volume. Two force plates (Bertec, Model MIE Ltd, Leeds, UK) were mounted within the laboratory floor and recorded ground reaction forces (1000 Hz). Anthropometric measurements were recorded for leg length, ankle width, knee width, height and mass. Thirty four markers (9 mm) mounted on rigid bases were attached to each participant’s right leg and foot by a single assessor (RM). A single marker set was created as an amalgamation of the marker sets for the three models (see Table 1): OFM [4], 3DFoot [8] and Kinfoot [7]. Only the position of marker 23 was compromised due to proximity of anatomical landmarks; the location between the second and third metatarsal head (in line with OFM placement) was chosen for all three foot models. The OFM uses the knee joint centre calculated from the Plug-in Gait (PiG) lower model to construct the tibial segment [3]. The location for all markers is listed in Table 1.

**Table 1:** Amalgamated marker set from the three foot models

**Segment**	**Marker number**	**Foot model**		
		**OFM**	**3DFoot**	**Kinfoot**
Shank /	1	Femoral condyle		
Tibia	2			Medial tibial condyle
	3			Lateral tibial condyle
	4	Tibial tuberosity	Tibial tuberosity	
	5	Head of fibular	Head of fibular	
	*6*	Lateral malleolus	Lateral malleolus	Lateral malleolus
	7	*Medial malleolus*	*Medial malleolus*	Medial malleolus
	*8*	Anterior aspect of the shin		Anterior tibia
Hindfoot	9	*Posterior distal heel*		
	10	Posterior proximal heel		
	11	Posterior calcaneus	Posterior calcaneus	Calcaneal tuberosity
	12	Lateral calcaneus	Peroneal tubercle	Anterior tubercle calcaneus
	13	Sustentaculum tali	Sustentaculum tali	Medial calcaneus
Talus	14			Lateral malleolus
				Medial malleolus
				Ankle joint centre (virtual)
	15			Second metatarsal centre (virtual)
Midfoot	16		Navicular tuberosity	
	17		Base of second metatarsal	
			Base of fifth metatarsal	
Cuboid				Calcaneus centre (virtual)
				Third metatarsal centre (virtual)
	18			Fifth metatarsal centre (virtual)
Forefoot	19	Base of first metatarsal	Base of first metatarsal	Base of first metatarsal (medial)
(Kinfoot presents	20		Base of second metatarsal	Base of third metatarsal (medial + lateral)
medial and	*21*	Base of fifth metatarsal	Base of fifth metatarsal	Base of fifth metatarsal (lateral)
lateral	22	*Head of first metatarsal*	Head of first metatarsal	Head of first metatarsal (medial)
Segments)	23	Between second and third metatarsal heads^A^	Head of second metatarsal	Head of third metatarsal (Medial + lateral)
	24	Head of fifth metatarsal	Head of fifth metatarsal	Head of fifth metatarsal (lateral)
Hallux	25	Base of hallux	Proximal phalanx of hallux	
	26			Origin of hallux triad on hallux nail
	27			Anterior of hallux triad
	28			Posterior of hallux triad
Toes	29			Origin of triad, hallux nail (medial)
	30			Head of first metatarsal (medial)
	31			Third metatarsal head (medial + lateral)
	32			Fifth metatarsal head (lateral)
	33			Third phalange nail (medial + lateral)
	34			Fifth phalange nail (lateral)

One row represents one marker, the descriptions of the anatomical landmarks are derived from the original manuscripts thus the description for each marker may vary. ^A^refers to the position chosen for Marker 23 for the amalgamated marker set as a compromise due to close proximity of anatomical landmarks. Marker number and names in *italics* refer to markers that were used for static trials only and were removed for dynamic trials.

Children were asked to ambulate barefoot at self-selected walking speed for three steps before entering the capture volume and three steps after. Six gait cycles of the right leg were captured and a retest interval of four weeks was implemented. Fourteen participants returned within the timeframe and data from three participants was excluded from further analysis.

### Data analysis

Three-dimensional marker trajectories were reconstructed using reconstruction parameters in Vicon software to ensure markers were visible during one complete gait cycle. Trajectories were gap filled to a maximum of three frames using a cubic-spline technique. Trials where subsequent trajectories were visible over the whole gait cycle were used for analysis. Gait events were determined by onset (initial contact) and conclusion (toe-off) of vertical force (20 N threshold) by the right foot on the force plate. At this point each trial was copied to make three identical trials before labelling and model processing according to each foot model’s protocol. To assess the repeatability of the joint angles for the three foot models data at specific events and peaks were extracted using ParamCalc (Vaquita Software, UK). Kinematic peaks from every segment of each foot model were taken as the absolute (no static offset was applied) maximal and minimal values during the stance and swing phase. The total number of variables extracted over the gait cycle was: 42 for OFM (seven segmental angles extracted at four peaks and two gait cycle events); 66 for 3DFoot (11 segmental angles extracted at four peaks and two gait cycle events); and 144 for Kinfoot (24 segmental angles extracted at four peaks and two gait events).

Spatiotemporal gait measures were averaged across 6 within-session trials. Anthropometric and mean spatiotemporal data were tested for significant differences between sessions by paired t-test or Wilcoxon non-parametric rank test. Within-rater repeatability of repeated angular values were measured by Intraclass correlation coefficients (3,*k*) [21]. Standard Error of Measurement (SEM) was calculated based on the ICC and pooled standard deviation. This gives units in degrees (°) which can be interpreted as the expected amount of error in repeated sessions. The equation for calculating SEM:

(1) $$\text{SEM}=\text{Sx}\sqrt{1-\text{rxx}}$$

Where S*x* is the pooled standard deviation (°) and r*xx* refers to intraclass correlation coefficient (ICC 3,*k*) [21,22].

To interpret ICC scores the scale proposed by Katz *et al*. [23] was used where ICC values > 0.80 represented very high, 0.60–0.79 moderately high, 0.40–0.59 moderate and < 0.40 low repeatability. In order to report the mean of multiple ICCs it was necessary to transform the *r* value by Fisher r-to-z transformation to take the average and transform this back to an ICC value (*r*). The authors adopted recommendations by McGinley *et al*. [24] where angular errors in excess of 5° could mislead clinical interpretation therefore errors less than 5° were deemed acceptable.

## Results

**Figure 1:**
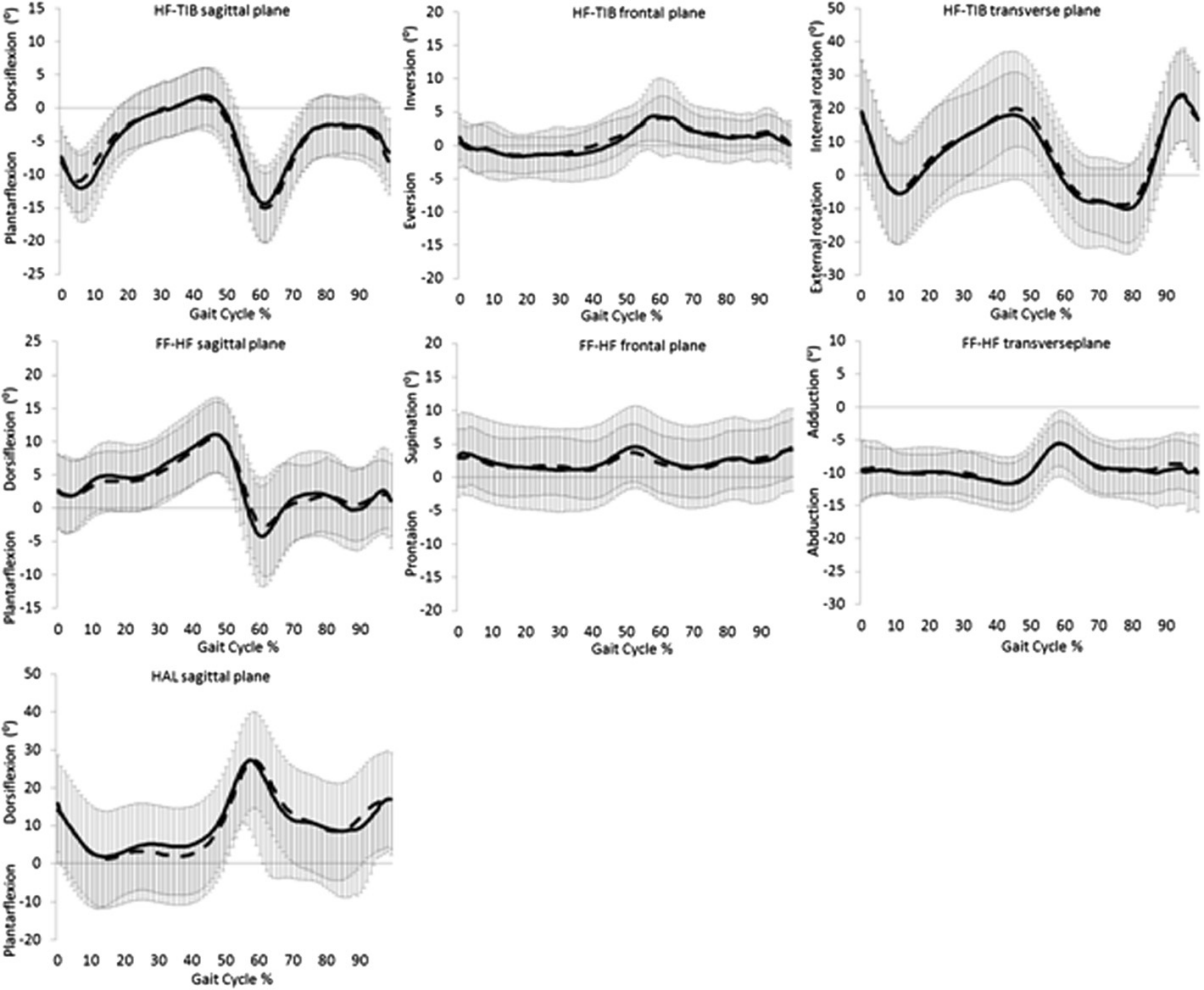
**Oxford foot model: mean (± SD) angular output.** Session 1 black line, session 2 dash line. HF = hindfoot, TIB = tibia, FF = forefoot, HAL = hallux.

**Figure 2:**
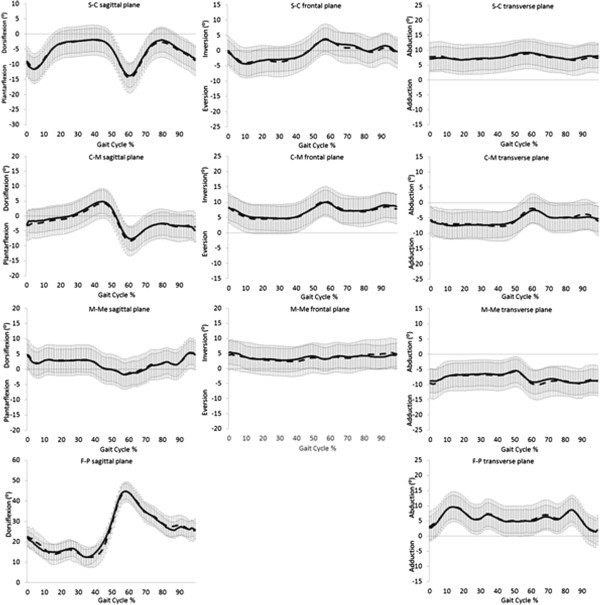
**3D foot: mean (± SD) angular output.** Session 1 black line, Session 2 dash line. S = shank, C = calcaneus, M = midfoot, Me = metatarsals, F = forefoot, P = phalanx.

**Figure 3:**
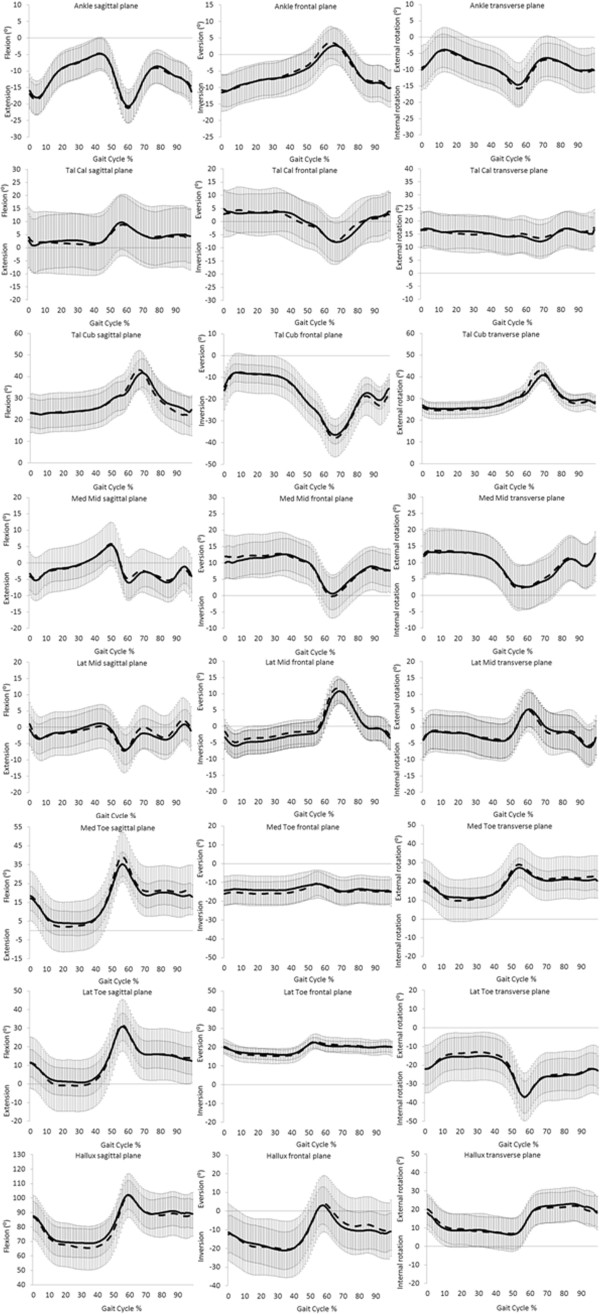
**Kinfoot: mean (± SD) angular output.** Session 1 black line, session 2 dash line. Tal = Talus, Cal = Calcaneus, Cub = cuboid, Med Mid = medial midfoot, Lat Mid = lateral midfoot, Med Toe = medial toe, Lat Toe = lateral toe.

Fourteen children with a mean age of 8.50 ± 2.79 years completed the study (see Table 2). There were no significant differences in anthropometric or spatiotemporal parameters between testing sessions (Table 2). Test-retest ICC and SEM values for the three models are presented (Tables 3, 4 and 5). All foot models demonstrated moderate repeatability (mean ICC 0.43 – 0.55). The OFM and 3DFoot models demonstrated acceptable mean error from repeated sessions (<5°) but Kinfoot demonstrated mean error >5°. Figures 1, 2 and 3 show representative OFM, 3DFoot and Kinfoot kinematic waveforms from session 1 and session 2 normalised to 100% of the gait cycle from the mean of all 14 participants.

**Table 2:** Mean anthropometric and spatiotemporal measures

	**Session 1**	**Session 2**	**Difference**	**Range**	***p*** **value between sessions**
Age (years)	8.50 ± 2.79	8.64 ± 2.84	0.14 ± 0.35	6 – 11	.165
Height (cm)	134.06 ± 36.46	134.94 ± 36.60	0.88 ± 0.01	123.0 – 154.5	.062
Body mass (kg)	31.00 ± 8.52	31.29 ± 8.73	0.29 ± 1.30	21.0 – 48.0	.104
BMI (kg/m^2^)	16.81 ± 4.92	16.87 ± 5.01	0.05 ± 0.36	13.57 – 21.43	.111
BMI Z-Score	0.15 ± 1.22	0.12 ± 1.26	0.030 ± 0.24	-2.11 – 2.38	.655
Leg length (cm)	70.07 ± 8.13	70.46 ± 8.33	0.39 ± 1.10	56 – 83	.097
Knee width (cm)	8.46 ± 0.63	8.53 ± 0.61	0.13 ± 0.68	6.4 – 9.6	.248†
Ankle width (cm)	5.95 ± 0.48	6.01 ± 0.45	0.06 ± 0.22	5.3 – 6.7	.347†
Cadence (steps/min)	131.82 ± 8.7	133.38 ± 11.4	1.56 ± 9.06	104.8 – 199.2	.530†
Stance phase (% of gait cycle)	57.54 ± 1.39	57.15 ± 1.28	0.39 ± 1.61	55.2 – 59.8	.420
Step length (m)	0.85 ± 0.24	0.93 ± 0.38	0.08 ± 0.20	1.15 – 2.21	.165
Step width (m)	0.15 ± 0.03	0.14 ± 0.03	0.0 ± 0.04	0.10 – 0.22	.825
Walking speed (m · s^-1^)	1.21 ± 0.15	1.23 ± 0.14	0.02 ± 0.14	0.93 – 1.55	.393

*p* represents the output of paired t-test or Wilcoxon non-parametric rank, significance set at <0.05. † refers to significance tested by Wilcoxon non-parametric rank test.

**Table 3:** Intraclass correlation coefficient and standard error of measurement of kinematic angles for Oxford foot model

**OFM**		**Gait cycle**					
**Segment**		**Initial contact**	**Stance min**	**Stance max**	**Toe-off**	**Swing min**	**Swing max**
HF-TIB sag	ICC (95% CI) (SEM°)	0.46 (0.06 to 0.73) 5.00	0.38 (-0.05 to 0.68) 1.79	0.67 (0.36 to 0.84) 2.99	0.47 (0.07 to 0.74) 2.92	0.41 (-0.02 to 0.69) 3.07	0.64 (0.31 to 0.82) 4.09
HF-TIB fro	ICC (95% CI) (SEM°)	0.62 (0.28 to 0.82) 2.18	0.47 (0.04 to 0.72) 2.92	0.51 (0.10 to 0.74) 2.46	0.14 (-0.29 to 0.52) 3.18	0.35 (-0.14 to 0.64) 2.83	0.43 (-0.03 to 0.69) 3.56
HF-TIB tra	ICC (95% CI) (SEM°)	0.75 (0.48 to 0.89) 9.77	0.68 (0.37 to 0.84) 7.74	0.76 (0.50 to 0.88) 6.90	0.80 (0.58 to 0.91) 8.81	0.83 (0.64 to 0.92) 6.00	0.69 (0.38 to 0.84) 8.38
FF-HF sag	ICC (95% CI) (SEM°)	0.65 (0.32 to 0.84) 4.42	0.54 (0.14 to 0.76) 4.13	0.68 (0.37 to 0.83) 3.35	0.54 (0.17 to 0.78) 4.24	0.65 (0.31 to 0.81) 4.12	0.65 (0.32 to 0.82) 4.72
FF-HF fro	ICC (95% CI) (SEM°)	0.46 (0.05 to 0.73) 5.18	0.57 (0.18 to 0.77) 3.12	0.62 (0.26 to 0.79) 2.80	0.61 (0.26 to 0.82) 3.82	0.60 (0.23 to 0.78) 2.75	0.61 (0.24 to 0.79) 4.06
FF-HF tra	ICC (95% CI) (SEM°)	0.45 (0.05 to 0.73) 3.66	0.70 (0.41 to 0.84) 2.32	0.51 (0.08 to 0.73) 3.36	0.49 (0.10 to 0.75) 2.99	0.54 (0.13 to 0.75) 3.09	0.41 (-0.08 to 0.67) 3.68
HAL sag	ICC (95% CI) (SEM°)	0.29 (-0.14 to 0.63) 11.25	0.45 (-0.03 to 0.69) 2.39	0.40 (-0.13 to 0.65) 11.77	0.34 (-0.08 to 0.66) 3.05	0.32 (-0.25 to 0.61) 2.99	0.29 (-0.29 to 0.59) 13.67

*HF*, Hindfoot, *Tib*, Tibia, *FF*, Forefoot, *HAL*, Hallux. *Sag*, Sagittal, *fro*, Frontal, *tra*, Transverse. Stance/Swing min = peak minimum angle during the stance/swing phase, Stance/Swing max = peak maximum angle during the stance/swing phase.

**Table 4:** Intraclass correlation coefficient and standard error of measurement of kinematic angles for 3DFoot

**3DFoot**		**Gait Cycle**					
**Segment**		**Initial contact**	**Stance min**	**Stance max**	**Toe-off**	**Swing min**	**Swing max**
S-C sag	ICC (95% CI) (SEM°)	0.81 (0.59 to 0.92) 2.65	0.66 (0.34 to 0.84) 3.63	0.74 (0.48 to 0.88) 3.55	0.74 (0.46 to 0.88) 4.12	0.58 (0.22 to 0.80) 4.25	0.55 (0.17 to 0.78) 4.80
S-C fro	ICC (95% CI) (SEM°)	0.41 (0.00 to 0.70) 4.56	0.65 (0.33 to 0.84) 3.10	0.32 (-0.11 to 0.65) 3.36	0.43 (0.02 to 0.71) 4.01	0.50 (0.11 to 0.76) 4.00	0.45 (0.04 to 0.73) 3.73
S-C tra	ICC (95% CI) (SEM°)	0.42 (0.01 to 0.71) 3.56	0.38 (-0.04 to 0.69) 2.47	0.54 (0.16 to 0.78) 2.10	0.58 (0.22 to 0.80) 2.95	0.40 (-0.01 to 0.70) 2.13	0.47 (0.08 to 0.74) 2.07
C-M sag	ICC (95% CI) (SEM°)	0.64 (0.31 to 0.83) 3.10	0.56 (0.19 to 0.79) 4.50	0.77 (0.52 to 0.90) 3.33	0.58 (0.22 to 0.8) 2.82	0.59 (0.24 to 0.81) 4.14	0.64 (0.32 to 0.84) 4.74
C-M fro	ICC (95% CI) (SEM°)	0.45 (0.05 to 0.73) 3.31	0.51 (0.12 to 0.76) 2.45	0.38 (-0.04 to 0.68) 2.36	0.24 (-0.19 to 0.59) 2.40	0.24 (-0.19 to 0.60) 2.43	0.31 (-0.12 to 0.64) 2.62
C-M tra	ICC (95% CI) (SEM°)	0.66 (0.34 to 0.84) 2.69	0.83 (0.64 to 0.93) 1.93	0.73 (0.46 to 0.88) 2.56	0.65 (0.33 to 0.84) 2.68	0.79 (0.57 to 0.91) 2.25	0.75 (0.49 to 0.89) 2.54
M-Me sag	ICC (95% CI) (SEM°)	0.23 (-0.20 to 0.59) 2.79	0.68 (0.38 to 0.86) 0.62	0.43 (0.02 to 0.72) 2.65	0.60 (0.24 to 0.81) 2.33	0.66 (0.35 to 0.85) 0.76	0.46 (0.06 to 0.74) 2.87
M-Me fro	ICC (95% CI) (SEM°)	0.40 (-0.02 to 0.69) 3.57	0.82 (0.61 to 0.92) 2.53	0.60 (0.25 to 0.81) 2.40	0.62 (0.27 to 0.82) 3.53	0.68 (0.38 to 0.86) 3.50	0.67 (0.36 to 0.85) 3.56
M-Me tra	ICC (95% CI) (SEM°)	0.45 (0.05 to 0.73) 2.67	0.59 (0.23 to 0.80) 2.61	0.63 (0.29 to 0.83) 2.87	0.55 (0.17 to 0.78) 3.53	0.42 (0.01 to 0.71) 2.95	0.50 (0.11 to 0.76) 3.54
F-P sag	ICC (95% CI) (SEM°)	0.40 (-0.02 to 0.69) 12.01	0.59 (0.23 to 0.81) 6.61	0.47 (0.07 to 0.74) 7.68	0.32 (-0.11 to 0.65) 13.86	0.36 (-0.06 to 0.67) 11.96	0.43 (0.02 to 0.71) 6.14
F-P tra	ICC (95% CI) (SEM°)	0.22 (-0.21 to 0.58) 6.94	0.21 (-0.22 to 0.57) 4.21	0.35 (-0.07 to 0.67) 5.11	0.24 (-0.19 to 0.59) 7.73	0.24 (-0.19 to 0.59) 4.58	0.08 (-0.34 to 0.48) 5.76

*S*, Shank, *C*, Calcaneus, *M*, Midfoot, *Me*, Metatarsals, *F*, Forefoot, *P*, Phalanx. *Sag*, Sagittal, *fro*, Frontal, *tra*, Transverse. *Stance/Swing min*, Peak minimum angle during the stance/swing phase, *Stance/Swing max*, Peak maximum angle during the stance/swing phase.

**Table 5:** Intraclass correlation coefficient and standard error of measurement of kinematic angles for Kinfoot

**Kinfoot**		**Gait Cycle**					
**Segment**		**Initial contact**	**Stance min**	**Stance max**	**Toe-off**	**Swing min**	**Swing max**
Ankle sag	ICC (95% CI) (SEM°)	0.48 (0.08 to 0.74) 3.69	0.49 (0.10 to 0.75) 3.62	0.49 (0.09 to 0.75) 2.91	0.42 (0.01 to 0.71) 4.36	0.42 (0.01 to 0.71) 4.50	0.58 (0.22 to 0.80) 3.39
Ankle fro	ICC (95% CI) (SEM°)	0.37 (-0.05 to 0.68) 3.60	0.47 (0.07 to 0.74) 2.60	0.54 (0.17 to 0.78) 2.56	0.65 (0.33 to 0.84) 3.27	0.49 (0.09 to 0.75) 2.79	0.6 (0.25 to 0.81) 4.00
Ankle tra	ICC (95% CI) (SEM°)	0.51 (0.13 to 0.76) 4.13	0.41 (-0.01 to 0.70) 3.39	0.60 (0.25 to 0.81) 3.86	0.46 (0.06 to 0.73) 4.50	0.45 (0.05 to 0.73) 4.09	0.31 (-0.12 to 0.64) 4.35
Tal Cal sag	ICC (95% CI) (SEM°)	0.60 (0.26 to 0.81) 7.30	0.68 (0.37 to 0.85) 6.79	0.64 (0.31 to 0.83) 6.58	0.68 (0.37 to 0.85) 6.70	0.54 (0.16 to 0.78) 8.98	0.58 (0.22 to 0.80) 7.12
Tal Cal fro	ICC (95% CI) (SEM°)	0.54 (0.17 to 0.78) 5.62	0.74 (0.46 to 0.88) 3.11	0.61 (0.26 to 0.81) 5.73	0.81 (0.60 to 0.92) 3.33	0.79 (0.55 to 0.91) 3.89	0.54 (0.17 to 0.78) 5.64
Tal Cal tra	ICC (95% CI) (SEM°)	0.46 (0.05 to 0.73) 5.01	0.46 (0.06 to 0.74) 3.85	0.52 (0.13 to 0.77) 4.36	0.65 (0.32 to 0.84) 3.52	0.60 (0.24 to 0.81) 3.45	0.49 (0.10 to 0.75) 4.28
Tal Cub sag	ICC (95% CI) (SEM°)	0.37 (-0.04 to 0.68) 6.28	0.26 (-0.17 to 0.61) 4.29	0.33 (-0.09 to 0.65) 3.78	0.32 (-0.11 to 0.65) 4.77	0.30 (-0.13 to 0.63) 4.57	0.48 (0.08 to 0.74) 8.64
Tal Cub fro	ICC (95% CI) (SEM°)	0.39 (-0.03 to 0.69) 5.54	0.49 (0.09 to 0.75) 5.97	0.31 (-0.11 to 0.64) 3.44	0.36 (-0.06 to 0.67) 8.43	0.52 (0.14 to 0.77) 13.21	0.34 (-0.09 to 0.66) 5.41
Tal Cub tra	ICC (95% CI) (SEM°)	0.23 (-0.20 to 0.59) 2.98	0.45 (0.04 to 0.72) 1.48	0.34 (-0.08 to 0.66) 2.13	0.46 (0.06 to 0.73) 2.82	0.40 (-0.02 to 0.70) 2.45	0.49 (0.10 to 0.75) 5.18
Med Mid sag	ICC (95% CI) (SEM°)	0.20 (-0.23 to 0.57) 4.92	0.34 (-0.09 to 0.66) 3.59	0.14 (-0.29 to 0.53) 2.65	0.34 (-0.08 to 0.66) 4.90	0.34 (-0.09 to 0.66) 2.75	0.34 (-0.09 to 0.66) 4.37
Med Mid fro	ICC (95% CI) (SEM°)	0.62 (0.28 to 0.82) 3.98	0.52 (0.14 to 0.77) 3.79	0.50 (0.11 to 0.76) 3.34	0.62 (0.27 to 0.82) 4.68	0.46 (0.07 to 0.74) 4.21	0.54 (0.17 to 0.78) 3.87
Med Mid tra	ICC (95% CI) (SEM°)	0.52 (0.14 to 0.77) 4.61	0.51 (0.13 to 0.76) 4.83	0.58 (0.22 to 0.80) 3.76	0.53 (0.15 to 0.77) 5.45	0.56 (0.19 to 0.79) 5.01	0.40 (-0.01 to 0.70) 2.67
Lat Mid sag	ICC (95% CI) (SEM°)	0.32 (-0.10 to 0.65) 4.67	0.40 (-0.02 to 0.70) 3.08	0.34 (-0.08 to 0.66) 2.83	0.29 (-0.14 to 0.63) 3.99	0.44 (0.04 to 0.72) 4.14	0.48 (0.08 to 0.74) 6.95
Lat Mid fro	ICC (95% CI) (SEM°)	0.51 (0.12 to 0.76) 2.12	0.55 (0.18 to 0.78) 2.12	0.43 (0.02 to 0.71) 2.40	0.33 (-0.09 to 0.65) 5.27	0.34 (-0.08 to 0.66) 2.76	0.61 (0.26 to 0.82) 11.08
Lat Mid tra	ICC (95% CI)	0.30 (-0.13 to 0.63) 5.01	0.51 (0.12 to 0.76) 3.38	0.52 (0.14 to 0.77) 4.93	0.42 (0.01 to 0.71) 6.46	0.43 (0.02 to 0.72) 4.26	0.55 (0.18 to 0.78) 5.71
Hallux sag	ICC (95% CI) (SEM°)	0.36 (-0.06 to 0.67) 10.23	0.59 (0.23 to 0.80) 5.99	0.37 (-0.05 to 0.68) 5.85	0.34 (-0.08 to 0.66) 8.68	0.39 (-0.03 to 0.69) 10.01	0.36 (-0.06 to 0.67) 6.35
Hallux fro	ICC (95% CI) (SEM°)	0.22 (-0.22 to 0.58) 7.37	0.43 (0.02 to 0.71) 4.30	0.23 (-0.20 to 0.59) 4.23	0.33 (-0.10 to 0.65) 5.62	0.48 (0.09 to 0.75) 6.01	0.19 (-0.24 to 0.56) 6.29
Hallux tra	ICC (95% CI) (SEM°)	0.58 (0.23 to 0.80) 5.76	0.58 (0.21 to 0.8) 4.15	0.69 (0.38 to 0.86) 4.89	0.43 (0.02 to 0.72) 5.60	0.44 (0.03 to 0.72) 4.15	0.76 (0.50 to 0.89) 4.16
Med Toe sag	ICC (95% CI) (SEM°)	0.34 (-0.09 to 0.66) 8.79	0.57 (0.21 to 0.80) 4.08	0.42 (0.01 to 0.71) 6.90	0.34 (-0.08 to 0.66) 8.93	0.08 (-0.34 to 0.48) 6.71	0.36 (-0.06 to 0.67) 7.19
Med Toe fro	ICC (95% CI) (SEM°)	0.58 (0.22 to 0.80) 4.61	0.70 (0.41 to 0.86) 3.65	0.53 (0.15 to 0.77) 5.26	0.49 (0.10 to 0.75) 5.67	0.49 (0.10 to 0.75) 5.31	0.52 (0.14 to 0.77) 5.21
Med Toe tra	ICC (95% CI) (SEM°)	0.52 (0.13 to 0.77) 6.46	0.41 (-0.01 to 0.70) 4.51	0.4 (-0.02 to 0.70) 5.23	0.29 (-0.14 to 0.63) 6.32	0.45 (0.05 to 0.73) 7.60	0.37 (-0.05 to 0.68) 5.44
Lat Toe sag	ICC (95% CI) (SEM°)	0.40 (-0.01 to 0.70) 8.82	0.44 (0.04 to 0.72) 4.88	0.32 (-0.11 to 0.65) 8.89	0.28 (-0.15 to 0.62) 10.31	0.27 (-0.16 to 0.62) 6.12	0.32 (-0.11 to 0.65) 8.63
Lat Toe fro	ICC (95% CI) (SEM°)	0.31 (-0.12 to 0.64) 2.94	0.39 (-0.03 to 0.69) 3.48	0.49 (0.09 to 0.75) 2.95	0.50 (0.11 to 0.76) 3.49	0.36 (-0.07 to 0.67) 2.82	0.44 (0.04 to 0.72) 3.11
Lat Toe tra	ICC (95% CI) (SEM°)	0.47 (0.07 to 0.74) 7.90	0.51 (0.12 to 0.76) 7.60	0.71 (0.42 to 0.87) 5.12	0.47 (0.07 to 0.74) 8.23	0.5 (0.11 to 0.76) 7.50	0.44 (0.03 to 0.72) 5.10

*Tal*, Talus, *Cal*, Calcaneus, *Cub*, cuboid, *Med Mid*, Medial midfoot, *Lat Mid*, Lateral midfoot, *Med Toe*, Medial toe, *Lat Toe*, lateral toe. *Sag*, Sagittal, *fro*, Frontal, *tra*, Transverse. *Stance/Swing min*, Peak minimum angle during the stance/swing phase, *Stance/Swing max*, Peak maximum angle during the stance/swing phase.

Repeatability of segmental kinematics across the two test sessions for the OFM were moderate with a mean ICC of 0.55 (95% CI 0.16 to 0.77) and reasonable mean SEM values of 4.61° ± 2.86°. The lowest ICCs for OFM segments were found at the hallux (ICC 0.35, 95% CI -0.15 to 0.63) where the SEM values (mean SEM 7.52° ± 5.23°) were also highest. Similarly, ICCs for inter-segmental kinematics of the 3DFoot were moderate with a mean ICC of 0.47 (95% CI 0.15 to 0.64) and reasonable SEM values (3.88° ± 2.18°). The lowest ICC for 3DFoot segments were reported at the hallux (mean of sagittal and transverse planes; ICC 0.31 95% CI -0.07 to 0.57) and high SEM values (mean SEM 7.72° ± 3.18°) were also found. Moderate ICCs were reported for Kinfoot (ICC 0.43, 95% CI -0.03 to 0.59) but mean SEM values were 5.08° ± 1.53°. Mean 3D rotations of the; subtalar (mean SEM 5.29° ± 1.69°), midfoot (mean SEM 5.08° ± 2.83°), hallux (mean SEM 6.09° ± 1.89°), medial toes (mean SEM 5.99° ± 1.50°) and lateral toes (mean SEM 5.99° ± 2.52°) demonstrated high levels of error.

Repeatability and test retest error of each foot model segment was assessed by determining the number of variables (defined as the angular values extracted for each 3D joint at gait cycle events and peaks) that were deemed to have low repeatability with high error. Table 6 presents the number of variables extracted from each foot model over the gait cycle that demonstrated low repeatability (ICC <0.4), and the number of variables that demonstrated unacceptable error (SEM >5°).

**Table 6:** Summary of mean repeatability, test- retest error and percentage of variables demonstrating low repeatability

**Foot model**	**Number of foot segments**	**Mean ICC (95% CI)**	**Percentage of variables with low ICC (%)**	**Mean SEM (**^**0**^**)**	**Percentage of variables with SEM > 5**^**0**^**(%)**
OFM	3	0.55 (0.16 to 0.77)	14.3	4.61 ± 2.86	26.2
3DFoot	4	0.47 (0.15 to 0.64)	22.7	3.88 ± 2.18	15.2
Kinfoot	8	0.43 (-0.03 to 0.59)	37.6	5.08 ± 1.53	43.8

### Hindfoot

The OFM demonstrated low repeatability for 3 of 42 (7.14%) variables at the hindfoot. 3DFoot demonstrated low repeatability for 2 of 66 (3.03%) hindfoot variables. Kinfoot also demonstrated low repeatability for 2 of 144 (1.39%) variables. 3DFoot demonstrated acceptable SEM values for all variables but SEM values of 7 (16.67%) OFM and 10 (6.94%) Kinfoot variables were high.

### Midfoot

The 3DFoot model demonstrated low repeatability in 4 (6.06%) midfoot variables. Kinfoot demonstrated low repeatability in 11 (7.64%) midfoot variables. SEM values were high for two (1.39%) Kinfoot variables, but for none of the 3DFoot variables.

### Forefoot

None of the forefoot variables for OFM demonstrated low repeatability. Low repeatability was found for 1 (1.52%) 3DFoot variable and for 12 (8.33%) Kinfoot variables. SEM values were greater than 5° in one (2.38%) OFM variable, no 3DFoot variables and seven (4.86%) Kinfoot variables.

### Hallux and toes

The OFM demonstrated low repeatability of four (9.52%) hallux variables. 3DFoot demonstrated low repeatability in eight (12.12%) variables and Kinfoot in nine (6.25%) hallux variables. SEM values exceeded the 5° in three (7.14%) OFM variables, 10 (15.15%) 3DFoot variables and 12 (8.33%) Kinfoot variables. Kinfoot’s toe segments demonstrated low repeatability in 13 (9.03%) and high SEM in 25 (17.36%) of variables extracted over the gait cycle.

## Discussion

With this study we sought to examine three foot models and establish their concurrent test-retest repeatability in evaluation of paediatric foot motion during gait. Motion of the hindfoot, midfoot, forefoot and hallux segments were analysed between the models across the gait cycle. All models demonstrated moderate overall repeatability but levels of repeatability and error varied across planes, segments and the gait cycle. Findings from this study were similar or less reliable than previous reports [4,7,18] and demonstrated low repeatability in the hallux across all three foot models, the hindfoot of Kinfoot and OFM and the midfoot of Kinfoot.

The hallux segment was consistently the least reliable (mean ICCs of 0.31-0.40) and demonstrated the highest levels of error (mean SEM of 6.09-7.52°). Repeatability of the hallux is likely to be affected by the close proximity of the phalanx markers leading to marker trajectory cross-over and drop out. For the OFM and 3DFoot, markers mounted on the medial hallux are used to define sagittal plane motion (OFM and 3DFoot) and transverse plane motion (3D Foot only). Kinfoot required a marker triad attached to nail of the hallux upon which two markers were attached, with a third on the base providing three markers for 3D motion tracking. This method of tracking the hallux may reduce marker trajectory cross-over and marker drop-out due a greater distance between triad markers compared to markers on the surface of the hallux. However, vibration artefact may be higher [7], increasing variability, and reducing any improvements in repeatability from greater inter-marker distances. Furthermore these findings may be reflective of more variable motion of the hallux during gait and further work is indicated.

Motion at the hindfoot segment for the OFM in the transverse plane demonstrated low repeatability and high error across repeated test sessions. This finding has been previously reported in OFM repeatability studies in children [4,17]. The transverse plane orientation of OFM’s hindfoot is dependent on the rotational alignment of the shank segment and the anterior axis of the hindfoot. Marker placement variability between the two sessions resulted in altered hindfoot orientation and therefore altered motion. Measurement of tibial torsion could be input into the model parameters to correct offset rotational malalignment of the shank. Variation in aligning the posterior calcaneal marker with the transverse plane of the hindfoot could result in lower reliability and higher errors between sessions. Curtis *et al.*[17] described the poor repeatability of OFM’s hindfoot in the transverse plane due to difficulty defining and identifying the neutral position of the hindfoot. The large intra-session standard deviation of the hindfoot transverse plane motion in the current study can be seen in Figure 1.

High error and low repeatability at the hindfoot was reported for Kinfoot at the subtalar and calcaneocuboid joints. Low repeatability for the subtalar and calcaneocuboid segments have previously been reported [7] and attributed to the estimated position of the talus/navicular/cuneiform and cuboid segments based on offsets from adjacent segments. When representing small segments in 3D, it may not be possible to attach three markers to bony landmarks. Therefore, Kinfoot presents two midfoot segments according to virtual markers where position is based on adjacent segment definitions. The talus/navicular/cuneiform and the cuboid segments are both oriented based on metatarsal segment position. High variability from assumptions of coupled motion between adjacent segments (subtalar and midfoot joints) and high variability from marker misplacement on the hallux and toes may be the causes of unacceptable error reported in this study.

A limitation of the protocol adopted was the compromise of marker placement on the forefoot segment. The 3DFoot model required a marker on the second metatarsal head, the OFM required a marker between the second and third metatarsal head and Kinfoot required a marker on the third metatarsal head. These locations were in too close proximity for three separate 9 mm markers to be attached to the skin. Therefore, the centre location, in-line with OFM, was chosen as a compromise. This may have induced errors in the orientation of 3DFoot and Kinfoot’s forefoot segments due to differences between the technical and anatomical coordinate systems. However, the compromised marker position did not appear to generate greater errors in the forefoot compared to other foot segments. Indeed, the amount of error in 3Dfoot and Kinfoot’s forefoot segments was consistent with previous findings [7,18]. It is possible that the compromised marker position was within the variability of marker placement found in the current study. Della Croce [25] found within-rater root mean squared differences of 9.0 mm when identifying the second metatarsal head which is the width of a marker used in the current study. Future work should consider examining the repeatability of these foot models in isolation as the close proximity of markers from three foot models may have reduced repeatability in the current study.

A second limitation of the protocol was the use of ICCs to describe the repeatability of discreet data points. Bland & Altman [26] stated that ICCs are dependent on the range of measurement across the population, with higher between-subject variation relative to total variation resulting in larger ICCs. This occurred in the OFM’s hindfoot segment which demonstrated greater between subject standard deviation (SD) relative to total SD increasing ICC values. The use of SEM has been recommended [24] as an absolute measure of error. This reduces interpretation of artificially high repeatability from ICCs by including inter-subject SD in the calculation. However, future research should consider other forms statistical analysis such as minimum levels of detectable change (MDC), minimally important differences (MCID) or limits of agreement (LoA) [24]. Gait cycle events as well as minimal and maximal peaks during the stance and swing phase were examined in this study. In clinical evaluation of foot motion, the focus of motion analysis may vary depending on the clinical presentation. Thus, information about the error values for foot segmental angles across the gait cycle may inform selection of appropriate foot models and interpretation of changes in foot motion in different populations. It is noteworthy that the repeatability of each foot model defined in this study is only directly applicable to a paediatric foot with no structural deformity. However, this information can aid decision making about the clinical utility of each foot model.

## Conclusion

The current study has examined three foot models and established their concurrent test-retest repeatability in evaluation of paediatric foot motion during gait. Findings from this work have demonstrated that segmental foot kinematics are repeatable in the paediatric foot but the level of repeatability and error varies across the segments. The OFM demonstrated moderate repeatability and reasonable errors in all segments except hindfoot motion in the transverse plane. Kinfoot offered an abundance of information on segment kinematics but repeatability was lower and errors higher than the accepted level. The 3DFoot model offered a balance of moderate repeatability and reasonable test-retest error similar to OFM, but with information on midfoot kinematics. Information on repeatability and expected test-retest errors of 3D foot models can better inform clinical assessment and the clinical utility of each foot model.

## Competing interests

The authors declare that they have no competing interests.

## Authors’ contributions

RM, SCM, WID and MCC all conceived and designed the study. RM collected and analysed the data. RM drafted the manuscript with the assistance of SCM, WID, and MCC. All authors approved the final manuscript.
